# Functionally validated imaging endpoints in the Alabama study on early age-related macular degeneration 2 (ALSTAR2): design and methods

**DOI:** 10.1186/s12886-020-01467-0

**Published:** 2020-05-19

**Authors:** Christine A. Curcio, Gerald McGwin, Srinivas R. Sadda, Zhihong Hu, Mark E. Clark, Kenneth R. Sloan, Thomas Swain, Jason N. Crosson, Cynthia Owsley

**Affiliations:** 1 Department of Ophthalmology and Visual Sciences, Department of Ophthalmology and Visual Sciences, School of Medicine, University of Alabama at Birmingham, 1720 University Blvd., Suite 609, Birmingham, AL 35294-0009 USA; 2 Department of Epidemiology, School of Public Health, University of Alabama at Birmingham, Birmingham, AL 35294 USA; 3 Doheny Eye Institute, P.O. Box 86228, Los Angeles, CA 90033 USA; 4 Department of Computer Science, College of Arts and Sciences, University of Alabama at Birmingham, Birmingham, AL 35294 USA; 5 Retina Consultants of Alabama, Birmingham, AL 35233 USA

**Keywords:** Age-related macular degeneration, Aging, Retina, Macula, Dark adaptation, Light sensitivity, Rods, Cones, Spectral domain optical coherence tomography, Quantitative autofluorescence

## Abstract

**Background:**

Age-related macular degeneration (AMD), a leading cause of irreversible vision impairment in the United States and globally, is a disease of the photoreceptor support system involving the retinal pigment epithelium (RPE), Bruch’s membrane, and the choriocapillaris in the setting of characteristic extracellular deposits between outer retinal cells and their blood supply. Research has clearly documented the selective vulnerability of rod photoreceptors and rod-mediated (scotopic) vision in early AMD, including delayed rod-mediated dark adaptation (RMDA) and impaired rod-mediated light and pattern sensitivity. The unifying hypothesis of the Alabama Study on Early Macular Degeneration (ALSTAR2) is that early AMD is a disease of micronutrient deficiency and vascular insufficiency, due to detectable structural changes in the retinoid re-supply route from the choriocapillaris to the photoreceptors. Functionally this is manifest as delayed rod-mediated dark adaptation and eventually as rod-mediated visual dysfunction in general.

**Methods:**

A cohort of 480 older adults either in normal macular health or with early AMD will be enrolled and followed for 3 years to examine cross-sectional and longitudinal associations between structural and functional characteristics of AMD. Using spectral domain optical coherence tomography, the association between (1) subretinal drusenoid deposits and drusen, (2) RPE cell bodies, and (3) the choriocapillaris’ vascular density and rod- and cone-mediated vision will be examined. An accurate map and timeline of structure-function relationships in aging and early AMD gained from ALSTAR2, especially the critical transition from aging to disease, will identify major characteristics relevant to future treatments and preventative measures.

**Discussion:**

A major barrier to developing treatments and prevention strategies for early AMD is a limited understanding of the temporal interrelationships among structural and functional characteristics while transitioning from aging to early AMD. ALSTAR2 will enable the development of functionally valid, structural biomarkers for early AMD, suitable for use in forthcoming clinical trials as endpoint/outcome measures. The comprehensive dataset will also allow hypothesis-testing for mechanisms that underlie the transition from aging to AMD, one of which is a newly developed Center-Surround model of cone resilience and rod vulnerability.

**Trial registration:**

ClinicalTrials.gov Identifier NCT04112667, October 7, 2019.

## Background

Age-related macular degeneration (AMD) is a leading cause of irreversible central vision impairment in the world [1]. It is a disease of the photoreceptor support system involving the retinal pigment epithelium (RPE), Bruch’s membrane (BrM), and choriocapillaris (ChC), in the setting of characteristic extracellular deposits between outer retinal cells and their blood supply. This pathology impacts retinoid re-supply, leading to photoreceptor demise and vision loss [2]. During the last several decades research has clearly documented the selective vulnerability of rod photoreceptors in the macula and rod-mediated (scotopic) vision during aging and early AMD as compared to cone photoreceptors and cone-mediated vision [3–11]. Several important findings lay down an innovative platform for our proposed research program. Rod-mediated dark adaptation (RMDA) is delayed in early AMD in that there is slowing in the recovery rate of light sensitivity after exposure to a bright light [6, 12]. Steady state rod-mediated light sensitivity, i.e., sensitivity to light after the retina adapts to a very low light level, is also impaired [5, 13–15]. We identified in a prospective study that delayed RMDA is the first-ever functional biomarker (risk factor) for incident early AMD [16]. Furthermore, we showed that those with high-risk ARMS2 and CFH sequence variants are more likely to have delayed RMDA [17]. RMDA also tracks progression through intermediate AMD in longitudinal studies, and it has been incorporated into large observational studies [18–20].

This report describes the recently-initiated Alabama Age-related Maculopathy Study 2 (ALSTAR2), a multi-disciplinary prospective study to establish a structural basis of RMDA delay and scotopic impairment in older adults at-risk for AMD and those who have already converted to early AMD. We will elucidate coincident structural and functional mechanisms underlying the transition from normal aging to AMD, and thus, mechanisms of disease initiation that will inform theories and models of early AMD pathogenesis. Results will also inform new endpoints for clinical trials evaluating early AMD treatments. In a large cohort of older adults who at baseline have either normal macular health or early AMD, we will assess structure using optical coherence tomography (OCT; spectral domain and OCT angiography), quantitative fundus autofluorescence (qAF), and scotopic function using RMDA and steady-state scotopic light sensitivity after adaptation to a dark background. Structure-function relationships will be examined cross-sectionally, topographically, and longitudinally, over 3 years. Our unifying hypothesis across all aims is that to the photoreceptors, early AMD is a disease of the retinoid re-supply route from plasma, due to detectable structural changes in the choriocapillaris-Bruch’s membrane-RPE complex (independent variables) which manifest functionally as delayed RMDA (dependent variable). In this model RMDA is also a measurable indicator for many molecular transfers occurring across this interface that if impaired may lead to additional metabolic and/or micronutrient insufficiencies.

The specific aims of ALSTAR2 are:

1. In AMD, the presence of extracellular deposits in the macula – soft drusen and subretinal drusen deposits (SDD, a.k.a reticular pseudodrusen) – increase AMD progression risk and compromise metabolic exchange between the ChC and outer retinal cells. Our hypothesis is that scotopic dysfunction will be accentuated where drusen and/or SDD are present, with SDD having a greater negative impact than drusen on scotopic function both cross-sectionally and prospectively.
2. RPE cell bodies lose epithelioid morphology before anterior migration or apoptosis, as evidenced by variability and thickening of the RPE band in OCT and dimming of fundus autofluorescence signal from organelles believed to contain bis-retinoids. The thickness of photoreceptor- and RPE-attributable reflective bands will be assessed for context. Our hypothesis is that variability in RPE cell bodies is associated more strongly with scotopic dysfunction than photopic function both cross-sectionally and prospectively.
3. The vascular density of the ChC (proportion of BrM covered) is a measure of direct metabolic exchange capacity between plasma and outer retinal cells in aging and early AMD. ChC vascular density declines in aging, under individual drusen, and in complete RPE and outer retinal atrophy (cRORA; geographic atrophy) [21]. Our hypothesis is that decreased ChC vascular density is more strongly associated with scotopic dysfunction than photopic function both cross-sectionally and prospectively.

### Public health significance

AMD is a complex, multi-factorial disease of aging in which central retinal photoreceptors are ultimately lost by a neovascular event or an atrophic process or both. About 14 million Americans have AMD, and this number is increasing. AMD generates significant personal burden due to reading and driving difficulties, depression, and anxiety [22–24]. Proven strategies for reducing AMD burden focus on preventing or stabilizing end-stage neovascularization with anti-vascular endothelial growth factor therapy, rightfully hailed as major scientific and medical breakthroughs [25–27]. Yet the vast majority of Americans with AMD have early disease. Currently there are neither proven means to arrest the progression of early AMD, nor prevention strategies for those at high risk.

A barrier to developing preventative and therapeutic strategies is a limited understanding of how aging, AMD’s largest risk factor, transitions to early AMD. Equally important is lack of valid and responsive endpoints acceptable to both the US Food and Drug Administration (FDA) and the scientific community [28, 29]. Anatomical (structural) endpoints are favored in clinical trials due to the speed, objectivity, and comprehensiveness of modern retinal imaging. Of these, the most promising will be those that correlate with visual function, facilitating use as primary outcome or surrogate measures. Thus, there is an urgent need to focus on precursors of early disease in order to understand the mechanisms underlying the earliest emergence of AMD and also to identify structural endpoints that can be functionally validated, i.e. structural and functional endpoints that are associated with each other.

### Background in physiology and aging

**Fig. 1:**
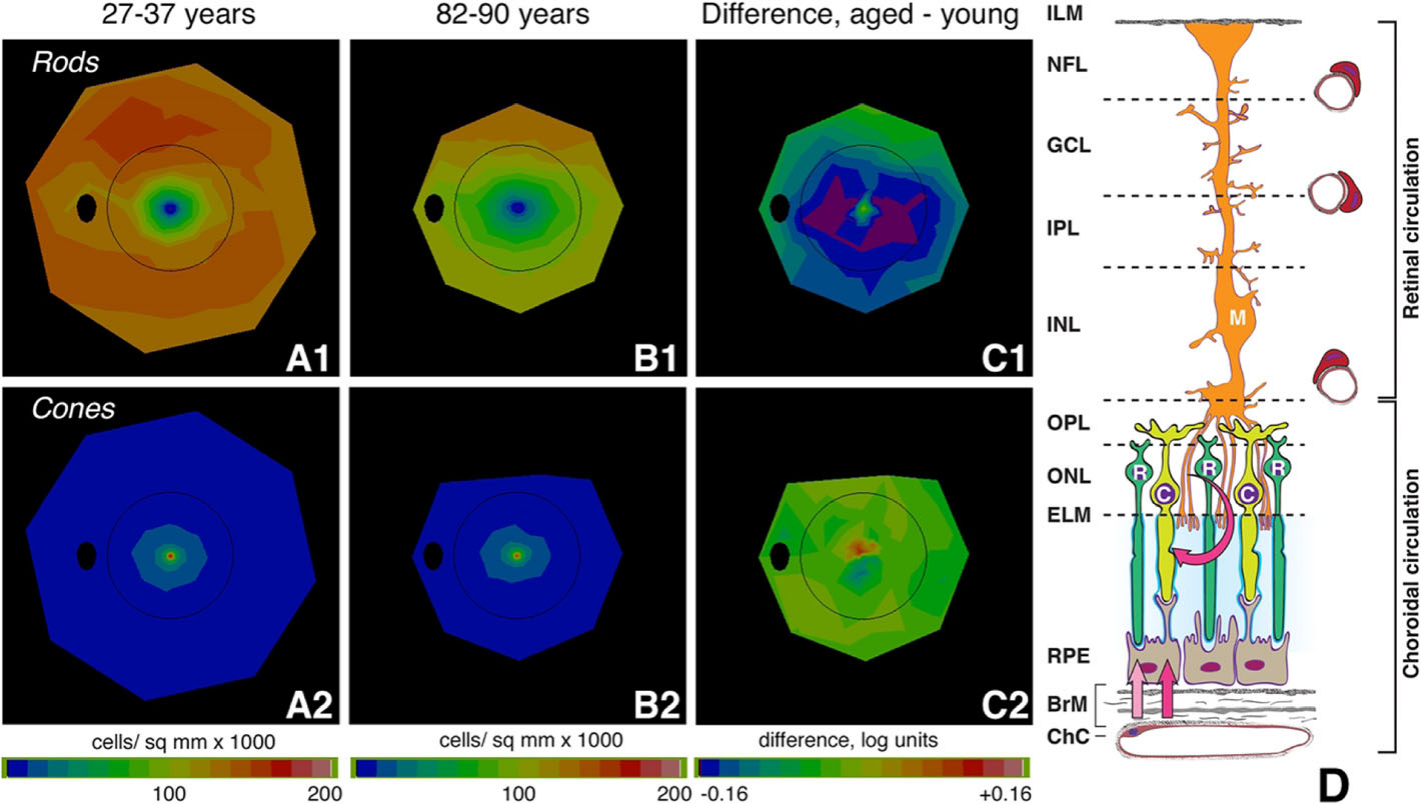
Aging of the human photoreceptor mosaic and the outer retinal neurovascular unit. A-C Rod vulnerability and cone resilience in healthy aging [34, 35]. Topography of rods and cones determined from computer-assisted cell counts in flat-mounts of human retina [3]. Maps are shown as a fundus of a left eye. Black oval, optic nerve; black ring, outer limit of Early Treatment of Diabetic Retinopathy Study grading grid. A1, A2. Rods and cones in 27–37-year-old donors. B1, B2. Rods and cones in 82–90-year-old donors. C1, C2. Log mean difference in cell density between younger and older adults. C1. Difference in rod density between younger and older adults is greatest at 0.5 mm to 3 mm from fovea (parafovea and perifovea). Purple signifies that aged eyes had 31% fewer cells than young eyes. C2. Log mean difference in cone density between younger and older adults is small and inconsistent, indicated by the yellow-green map. D Outer retinal neurovascular unit and retinoid re-supply to cone and rod photoreceptors. Shown are rods (R), cones (C), Müller glia (M), RPE, and vascular endothelium of the choriocapillaris (ChC) and capillary plexuses of the retinal circulation. We hypothesize that to the photoreceptors, AMD is a disease of the retinoid re-supply route. Vitamin A delivered from plasma is rate-limiting for recovery of sensitivity by rods. Rods need choriocapillaris, Bruch’s membrane, and RPE, whereas the cones have these, plus an additional second delivery route, via Müller glia and the retinal circulation. RMDA assesses how pathology in the choriocapillaris, Bruch’s membrane, and RPE complex impacts rods. It is expected that cone-mediated vision will be resilient. Retinal layers: ILM, inner limiting membrane; NFL, nerve fiber layer; GCL, ganglion cell layer; IPL, inner plexiform layer; INL, inner nuclear layer; OPL, outer plexiform layer; ONL, outer nuclear layer; ELM, external limiting membrane; RPE, retinal pigment epithelium; BrM, Bruch’s membrane; ChC, choriocapillaris

The human retina is overall dominated by rod photoreceptors over cones (rod:cone ratio, 21:1). Because AMD’s debilitating vision loss affects central vision, we emphasize that the macula is also rod-dominated, despite the presence of the fovea, according to histologic studies using computer-assisted flat mounts to ensure accurate and unbiased counts [3, 30] and then validated in vivo with single-cell imaging [31]. “Macula” is variably defined, and Table 1 shows photoreceptor content in subfields of the Early Treatment of Diabetic Retinopathy Study (ETDRS) grading grid, widely used to assess AMD prevalence, incidence, and severity by color fundus photography. In this grid, rods outnumber cones 8.7:1 in younger adults and 6.8:1 in younger adults. The central 1 mm diameter subfield contains the cone-only foveola (0.375 mm in diameter) and a few rods around its rim, accounting for the non-zero rod: cone ratio in Table 1. In younger and older adults, respectively, mean rod density is 4.2-fold and 3.2-fold higher than cones in the inner ring (para- and perifovea), and 12.9-fold and 10.1-fold higher in the outer ring (perifovea) [32, 33]. These studies showed that in aging macula, rods die before cones, in a parafoveal ring directly surrounding the fovea, where loss is 30% up to age 92 (Fig. 1a-c). Further, histology also revealed that the last photoreceptors surviving in late AMD are cones [3, 4]. A monotonic trajectory of greater loss of rods than cones, beginning in aging, is a parsimonious explanation for these data. It is important to note that the highest densities of rods are outside the ETDRS grid in an elliptical ring at 2–5 mm eccentricity and extending into the nasal retina. The principal signal source for FAF imaging (Aim 2) is RPE lipofuscin, long-lasting inclusion bodies within these cells, and this tracks photoreceptor topography precisely, with highest intensities overlying the rod ring [36]. Thus, parafoveal rod loss in aging is spatially uncorrelated with highest levels of lipofuscin-attributable autofluorescence, as noted [3].

**Table 1:** Photoreceptor density (cells/ mm^2^) in human macula [3, 30]

ETDRS subfields (inner to outer radius in mm and degrees of visual angle)	Mean cells/ mm^2^ in subfield		Ratio of Rods to Cones
	Cones/ mm^2^	Rods/ mm^2^	Rods : Cones
	Ages 27-37 years		
Center (0 - 0.5 mm; 0 - 1.7°)	57,001	20,013	0.35
Inner (0.5 - 1.5 mm; 1.7 - 5.2°)	18,774	78,252	4.17
Outer (1.5 - 3.0 mm; 5.2 - 10.4°)	10,138	130,750	12.90
Mean photoreceptor density in ETDRS grid	13,354	116,037	8.69
	Age 61-90 years		
Center (0 - 0.5 mm; 0 - 1.7°)	54,897	15,016	0.27
Inner (0.5 - 1.5 mm; 1.7 - 5.2°)	17,673	56,105	3.17
Outer (1.5 - 3.0 mm; 5.2 - 10.4°)	9,512	96,265	10.12
Mean photoreceptor density in ETDRS grid	12,582	85,106	6.76
**RMDA test spot locations**	Ages 27-37 years		
5° superior	15,530	82,357	5.30
12° superior	9,406	145,683	15.49
	Ages 61-82 years		
5° superior	15,376	62,043	4.04
12° superior	8,835	116,683	13.21

1 mm = 3.5° visual angle

The ETDRS grid is 28.27 mm^2^ in area and contains 377,530 cones and 3,280,371 rods in retinas of donors aged 27-37 and 355,678 cones and 2,405,952 rods in retinas from donors aged 61-90. Densities at specific locations were determined by finely space resample of the original data

*ETDRS* Early Treatment of Diabetic Retinopathy Study, *RMDA* Rod-mediated dark adaptation

RMDA assesses the efficiency of replenishing retinoids and loading of 11-*cis*-retinal to the opsin molecule in rod outer segments, as established by a landmark quantitative analysis [37–39] incorporating fundus reflectometry, visual cycle biochemistry, and clinical observations of sensitivity recovery in outer retinal disease. The visual cycle is the process of eliminating products of light absorption from outer segments, recycling of released retinoid to its original form (11-*cis*-retinal), and regenerating the visual photopigment opsin [37, 38]. The RPE serves photoreceptors in many ways, including the delivery of fresh 11-*cis*-retinal, uptake and translocation of nutrients and hormones, phagocytosis of spent OS tips, and elimination of unneeded metabolites. Cones are also served by the RPE and choriocapillaris, but due to a second visual cycle (in Müller glia), which depend on retinal circulation [40, 41], they are less vulnerable to ChC-BrM-RPE pathology than are rods, at least in early AMD stages. On the basis of our prior prospective study (ALSTAR 1), we hypothesize that to the photoreceptors, AMD is a disease of the retinoid re-supply route to rods, directly implicating changes in essential structures external to the outer segments (ChC endothelium, BrM, RPE; Fig. 1d).

**Fig. 2:**
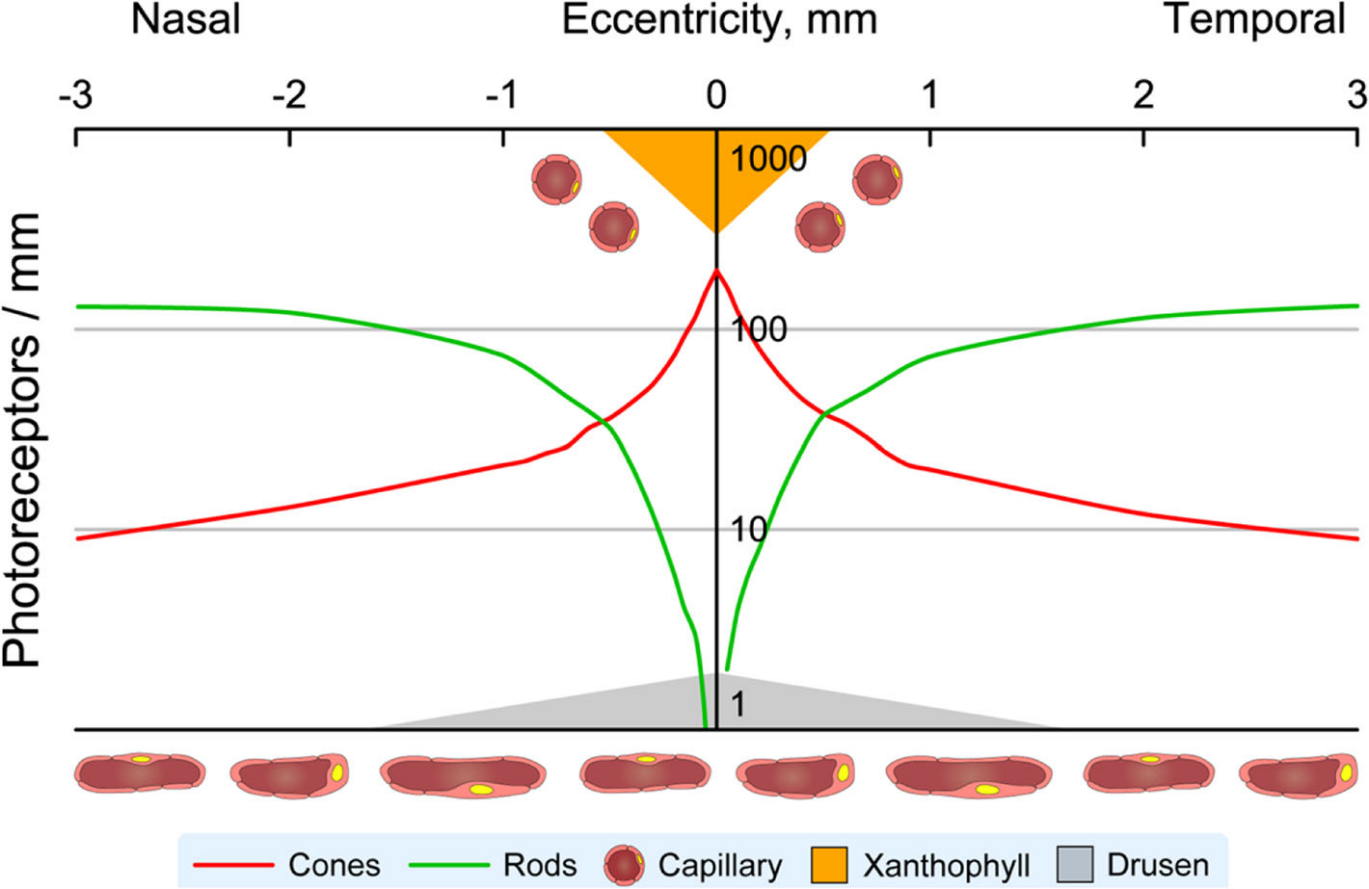
Visual neuroscience of human retinal aging and age-related macular degeneration.Schematic shows log cone and rod density [30], choriocapillaris and retinal vessels, and helpful/ harmful factors for photoreceptor function and survival in the human macula. Soft drusen and basal linear deposits (gray) accumulate between choriocapillaris and retinal cells. They are thickest and confer highest risk for progression in central macula. Xanthophyll carotenoid pigment (orange) is highest in the foveal center (shown) with lateral extensions into the plexiform layers (not shown), plausibly attributable to the distribution of protective Müller glia [34]. The retinal capillaries and choriocapillaris are shown

A strong point of the ALSTAR2 design is the incorporation of new concepts in outer retinal physiology and importantly, the biology of soft drusen, the earliest discovered and largest risk factor for progression to AMD end stages (odds ratio for progression risk at 10 years = 26) [42–45]. The vertically organized and tightly integrated physiologic unit of photoreceptors, Müller glia, RPE, ChC, and deep capillary plexus can be considered the outer retinal neurovascular unit (Fig. 1d) [46]. This construct, developed originally for brain and then inner retina [47, 48] comprises micro-vessels, neurons, glia, pericytes, and extracellular matrix that couple blood flow to the metabolic demands of neurons. AMD can be conceived as either a neurodegeneration or a disease of vascular/metabolic insufficiency, depending on the initial site of damage. Marked histologic aging alterations in human retina are the buildup of a lipid-rich barrier in macular BrM and a decline in ChC vessel density and endothelial functionality throughout adulthood [49–52], which together result in the formation and growth of soft drusen centrally (Fig. 2). Drusen grow, because the RPE constitutively produces apolipoprotein B,E-containing lipoproteins and other material that normally exits to the circulation but is impeded in transit by aged BM-ChC. This “Oil Spill” hypothesis has compelling support from longitudinal clinical imaging and cell culture studies [53, 54]. ALSTAR2 study design explores this hypothesis by comparing structures and functions attributable to different layers of the neurovascular unit in aged normal eyes and in early AMD.

Our vascular-metabolic model, because it is linked to the topography of retinal neurons, can be contrasted with (but does not exclude) mechanisms of AMD progression that have a pan-retinal or systemic basis (e.g., oxidative stress, inflammation, complement activation) [55, 56]. If our hypotheses withstand rigorous testing, strategies to maintain or restore health of ChC endothelium and/or BrM may have merit in protecting neurons, a winning strategy for tackling cerebrovascular disease and stroke. Our research plan, described below, seeks to identify specific changes in chorioretinal anatomy that are structural correlates for scotopic dysfunction including delayed RMDA. At the same time, we will also test cone-mediated vision to probe cone resilience and concurrently acquire specialized autofluorescence images for visualizing macular pigment (see Discussion).

### Conceptual framework for study design

**Fig. 3:**
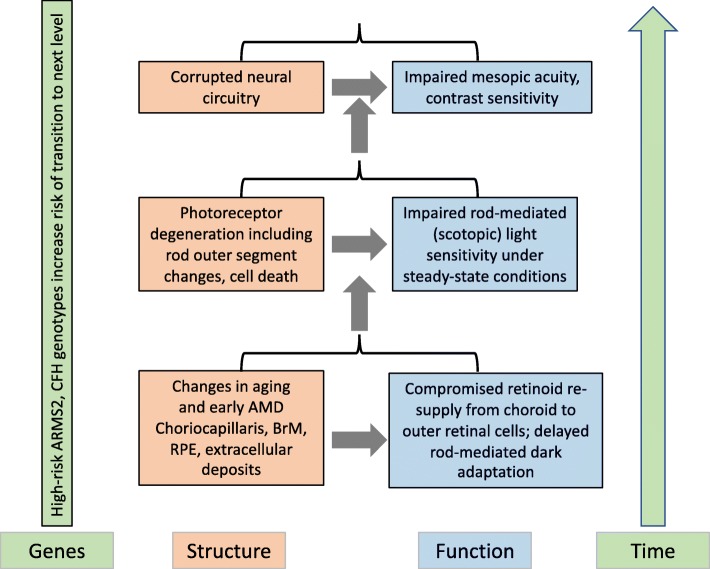
Conceptual framework of visual impairment across retinal layers and time. Outer retinal neurovascular is at the bottom of the framework. Inner retina is at the top

The ALSTAR2 study design is based on the conceptual framework depicted in Fig. 3. As aging transitions to early AMD, our framework focuses on three factors - retinal structure, visual function, and genes – and three partly overlapping epochs in time. As depicted by the gray area in Fig. 3, the first longitudinal epoch in time addresses the aging process and the relationship between structure and RMDA, a measure of retinoid re-supply. The second epoch addresses the relationship between structure and scotopic sensitivity as aging transitions into early AMD. The third epoch addresses the transition from early to intermediate AMD and the relationship between mesopic acuity and contrast sensitivity at fixation, which are heavily impacted by corrupted neural retinal circuitry. We hypothesize that high-risk variants in the AMD susceptibility genes *ARMS2* and *CFH* increase the likelihood and rate of moving from one epoch to the next.

In Fig. 3, we hypothesize that RMDA will be the earliest visual dysfunction in the aging to early AMD transition because of its dependence on the canonical retinoid re-supply route from ChC. As mentioned earlier, cones have an alternative source of retinoids. Thus, RMDA is more vulnerable to slowing than is cone-mediated dark adaptation, although we do not expect cones to be unaffected. We also hypothesize that as early AMD emerges (second epoch, Fig. 3) with rod photoreceptor outer segments shortening and degenerating, steady state scotopic (rod-mediated) light sensitivity will decrease. Steady state sensitivity refers to light sensitivity after the retina has ample time to adapt to the background luminance level of the target. Ultimately, as rods degenerate and die, retinal signal transmission and retinal circuitry (e.g., connections between rods and bipolar cells) will be corrupted further generating the formation of aberrant inner retinal connections [57–59] (third epoch, Fig. 3). This will result in deficits in mesopic central visual acuity and spatial contrast sensitivity at fixation, which are mediated by both rods and cones. ALSTAR2 is an opportunity to determine which types of rod dysfunction emerge before others. In addition, changes in cone-mediated vision can be directly compared to and contrasted with changes in rod-mediated vision in the same patients, an experimental paradigm that is seeing success in animal model studies [60].

In addition to examining structural and functional interrelationships in the aging to early AMD transition, ALSTAR2 will also reveal the timeline of several rod-mediated dysfunctions in aging and early AMD for insight into the neurophysiology of affected cells. The temporal relationship between RMDA delay, steady-state scotopic sensitivity loss, and mesopic central vision impairment in early AMD has not been established. We hypothesize that delayed RMDA will precede or possibly coincide with impaired scotopic sensitivity. Conversely, impaired scotopic sensitivity will not precede delayed RMDA. This hypothesis has preliminary support from cross-sectional studies but has not been examined longitudinally [6, 10, 61]. A second hypothesis is that delayed RMDA and scotopic sensitivity impairment will both precede deficits in mesopic visual acuity (mediated by both rods and cones) and central photopic contrast sensitivity (mediated by cones), measured at fixation. Cross-sectional studies have shown that eyes with early AMD can have modest impairments in mesopic visual acuity and contrast sensitivity, and these deficits can be more serious in intermediate AMD [62–67]. Further, impaired mesopic visual acuity and a low luminance deficit (disproportionate loss of mesopic acuity compared to photopic acuity) is associated with conversion to geographic atrophy, a form of advanced AMD [68].

## Methods/design

### Study design

The study was approved by the Institutional Review Board of the University of Alabama at Birmingham (UAB). All enrollees will provide written informed consent after the nature and purpose of the study are explained. Conduct of the study follows the tenets of the Declaration of Helsinki.

ALSTAR2 is a prospective cohort study with baseline measurements that are repeated at follow-up 3 years later. At baseline two groups of adults ≥60 years old are enrolled whose AMD status is determined by the AREDS 9-step classification system for color fundus photographs [69, 70]: (1) Those in normal macular health (AREDS grade 1) in both eyes, i.e., drusen equivalent diameter < 125 μm without increased pigmentation or depigmentation/ GA. (2) Those having early AMD (AREDS grade 2–4) in at least one eye, i.e. different combinations of drusen abundance and pigmentary change [69, 71]. The fellow eye can be any AREDS grade. We selected a 3-year follow-up period since changes in structural and functional characteristics in aging and early AMD are detectable over this period [16, 18, 19, 72]. We chose to use the AREDS 9-step grading system, despite the limitations of color fundus photography in revealing important details of AMD pathology, because it is validated and widely used [73, 74], and an equivalent system for OCT or other technology is yet to be devised. However, key pathology like SDD is visible in good color fundus photographs [75, 76] and are amenable to deep learning approaches [77] for detection and staging.

Exclusion criteria for those in normal macular health are (1) any eye condition or disease in either eye (other than early cataract) in the medical record that can impair vision including diabetic retinopathy, glaucoma, ocular hypertension, history of retinal diseases (e.g., retinal vein occlusion, retinal degenerations), optic neuritis, corneal disease, previous ocular trauma or surgery, refractive error ≥ 6 diopters; (2) neurological conditions that can impair vision or judgment including multiple sclerosis, Parkinson’s disease, stroke, Alzheimer’s disease, seizure disorders, brain tumor, traumatic brain injury; (3) psychiatric disorders that could impair the ability to follow directions, answer questions about health and functioning, or to provide informed consent; (4) diabetes; (5) any medical condition that causes significant frailty or is believed to be terminal. Exclusion criteria for the early AMD group are identical to those described above, except that it is acceptable for participants to have early AMD (AREDS 2–4) in one eye and be AREDS grade 1 or any stage of AMD in the fellow eye.

### Setting and protocol

ALSTAR2 is being conducted in the Clinical Research Unit of the Department of Ophthalmology and Visual Sciences at the University of Alabama at Birmingham, based at the Callahan Eye Hospital. Participants are recruited through the comprehensive eye care and retina clinics at the Callahan Eye Hospital. Participants from the first ALSTAR study [16, 71] are eligible to participate if they meet inclusion and exclusion criteria.

Table 2 lists assessments conducted in the protocol. All protocol elements are collected at baseline and then repeated at follow-up 3 years after baseline (with the exception of a blood sample for DNA extraction). Because of the length of the protocol, both baseline and follow-up consist of two separate visits within 1 week of each other, which together last approximately 4–6 h. Tropicamide 1% and phenylephrine hydrochloride 2.5% are used to dilate pupils (diameter of ≥6 mm) as needed for specific parts of the protocol.

**Table 2:** Protocol assessments

Assessments	
**Visual Function Tests**	
Rod-mediated dark adaptation	
2-color dark-adapted microperimetry	
Light-adapted perimetry	
Photopic acuity	
Mesopic acuity	
Photopic contrast sensitivity	
Mesopic contrast sensitivity	
**Multi-Modal Retina Imaging**	
Color fundus photography	
Color fundus photography	
Near-infrared reflectance	
OCT-angiography	
Quantitative autofluorescence	
**Laboratory Assays**	
High density lipoprotein	
C-reactive protein	
DNA extraction and gene screening	
**Questionnaires**	
Demographics	
General health	
Medication use	
Alcohol use	
Smoking	
Low Luminance Questionnaire	
Vision in Low Luminance	
Montreal Cognitive Assessment	

Visual functional tests include tests of rod function, cone function and mixed rod and cone (mesopic) function. RMDA will be measured by the AdaptDx (MacuLogix, Harrisburg PA). Testing occurs in a dark, light-tight room, after dilation. RMDA will be measured at 5° (where rod loss is maximal in aging and AMD) and 12° (where rod density is maximal and rod loss is disproportionately less) [3, 4]. At 5° and 12° respectively, the rod-cone ratio is 8.5 at 5° and 23.2 at 12° in young adults and 6.3 and 18.3 in older adults (Table 1). RMDA will be tested in one eye only to minimize participant burden (i.e., in the eye with better acuity). The order of RMDA testing for the 5° and 12° targets will be counterbalanced across subjects. The procedure begins with a photo-bleach exposure to a 6° flash centered at the test target location on the superior vertical meridian on the retina (equivalent ~ 83% bleach; 0.25 ms duration, 58,000 scotopic cd/m^2^ s intensity [78]) while the participant focuses on the fixation light. Threshold measurement (3-down/1-up threshold strategy) for a 2° diameter, 500 nm circular target begins 15 s after the bleaching offset. The participant is instructed to maintain fixation and press a button when a flashing target first becomes visible within the bleached area. Log thresholds are expressed as sensitivity in decibel (dB) units as a function of time since bleach offset. Threshold measurement continues at 30-s intervals for 20 min, or until the rod-intercept time (RIT) can be estimated. RIT is the duration in minutes required for sensitivity to recover to a criterion value of 5.0 × 10^− 3^ scotopic cd/m^2^ [16, 79], located in the latter half of the second component of rod-mediated recovery [37, 80]. RIT is largely influenced by the rate of rhodopsin generation [37, 38, 81–85] and has good test-retest reproducibility (*r* = 0.95) [86]. RIT at the 5° and 12° test locations will also be compared within-subject to assess a parameter called “Rod Slope” [61].

**Fig. 4:**
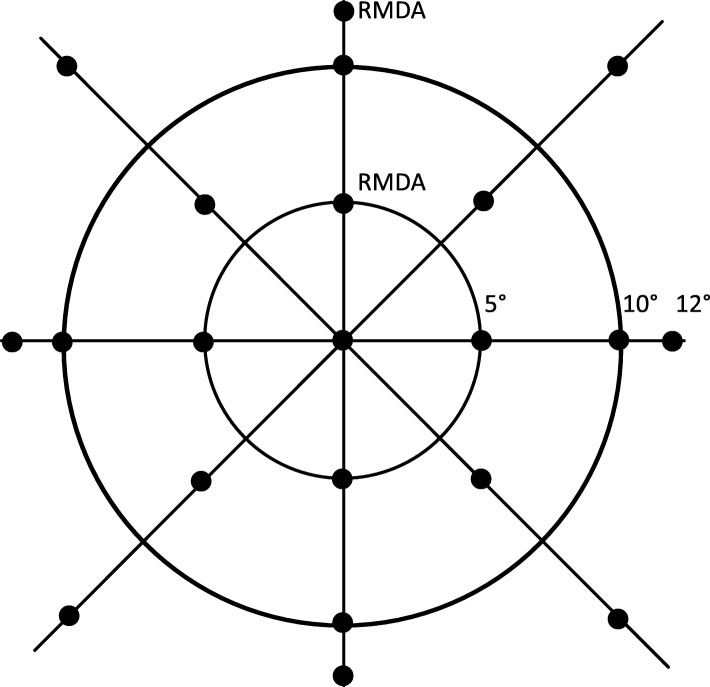
Test locations for perimetry. The same 21 locations will be used for 3 test paradigms, the Humphrey Field Analyzer (for photopic sensitivity) and two-color dark-adapted microperimetry (cyan 505 nm, red 627 nm) using the MAIA-S. Perimetry will be tested in one eye only (the eye undergoing testing for RMDA tested at 5° and 12°). Targets are 0.43° in diameter (Goldmann III). Locations were chosen to sample closely near the fovea, where cell densities change rapidly with eccentricity and an effect of Bruch’s membrane lipidization is expected. In addition, targets in perifoveal and paramacular locations are included where rod density is high and subretinal drusenoid deposit is expected

Two-color dark-adapted microperimetry is a method that facilitates determining whether light sensitivity measurements are rod-mediated, cone-mediated, or have mixed mediation (both rods and cones) [87, 88]. Microperimetry will be tested in one eye only to minimize participant burden (the same eye tested for RMDA). After participants adapt to darkness for 30 min, the Scotopic MAIA (S-MAIA) microperimeter (CenterVue, Fremont CA) is used to measure light sensitivity in dB for a grid of 23 test targets presented in the macula (Fig. 4**)**. Targets are 0.43° in diameter (size Goldmann III); testing is carried out for both cyan (505 nm) and red (627 nm) targets after dilation. This “two-color” approach under dark adapted conditions is a well-established method in inherited retinopathy research [87, 88] and will verify whether sensitivity for the cyan target is indeed rod-mediated and not subject to cone intrusion. Thresholds not meeting the rod-mediated criterion are excluded from the characterization of scotopic sensitivity. Outcome measures will include both a global sensitivity across the macula and a regional or focal sensitivity, for specific eccentricities, meridians, and in correspondence to retinal structure.

Since cone-mediated sensitivity in the visual field is optimized for photopic light conditions, we will also assess sensitivity using a Humphrey Field Analyzer with a full-threshold procedure for a test grid identical to that of the S-MAIA as indicated above. The same eye as tested for dark adaptation and S-MAIA testing will be used.

Best-corrected visual acuity and spatial contrast sensitivity will be measured under both photopic (100 cd/m^2^) and mesopic (0.1 cd/m^2^) conditions for each eye separately. Visual acuity will be assessed using the Electronic Visual Acuity (EVA) tester [89] (JAEB Center, Tampa FL) and expressed as logarithm_10_ of the minimum angle of resolution. To achieve background mesopic luminance, participants will view the EVA through a 2.0 log unit neutral density filter. The “low luminance deficit (LLD)” for acuity [62], as it is called, is defined as the decrease in logMAR that occurs under mesopic conditions as compared to acuity under photopic conditions. Analogous to measuring LLD for acuity, we will assess the LLD for contrast sensitivity by measuring contrast sensitivity under both photopic and mesopic conditions as described above, using the Mars chart [90] (Mars Perceptrix, Chappaqua NY). The LLD for contrast sensitivity will be expressed as the difference in logarithm_10_ of sensitivity under photopic versus mesopic conditions. Both the EVA visual acuity tester and the Mars contrast sensitivity chart have high test-retest reproducibility [89, 91].

With respect to multi-modal imaging, protocol elements consist of color fundus photography (CFP), SDOCT, near-infrared (NIR) reflectance, OCT angiography, and quantitative autofluorescence (qAF). OCT angiography is captured on the study eye only (eye tested for RMDA). Other modalities are captured on both eyes. CFP is used to document eligibility and AMD status. After pupil dilation, 3-field (30° diameter) stereo-fundus, digital photographs using a FF450^+^ camera (Carl Zeiss Meditec, Dublin CA) are taken. Photos will be evaluated by a trained grader with established inter-grader agreement (κ = 0.84) for presence and severity of AMD using the AREDS 9-step classification system [69]. The AREDS system grading is also converted to the more recently developed Beckman system [92]. Inter-grader agreement will be periodically monitored using a second grader with disagreements adjudicated by a fellowship-trained retina specialist (JNC). In addition, with the FF450+ camera, CFPs are captured at 2 additional magnifications (20° and 50°).

We acquire spectral-domain OCT volumes (Spectralis HRA + OCT, Heidelberg Engineering, Heidelberg, Germany; λ = 870 nm; scan depth, 1.9 mm; axial resolution, 3.5 μm per pixel in tissue; lateral resolution, 14 μm per pixel in tissue), with Automatic Real-Time averaging > 9, and quality (signal-to-noise) 20–47 dB. B-scans (*n* = 121 scans, spacing =60 μm) are horizontally oriented and centered over the fovea in a 30°× 25° (8.6 × 7.2 mm) area. To permit averaged OCT-angiography scans of the choriocapillaris [93], we take two volumes for OCT-angiography centered at each of two locations in the study eye, the fovea and the 5° RMDA test location. Both of these cover 15°× 15° (4.3 × 4.3 mm, *n* = 384 scans, spacing = 11 μm). To detect SDD, which extend not only beyond typical SDOCT volumes but also outside the vascular arcades and nasal to the optic nerve head [94, 95], we use 50° *en face* fundus imaging with CFP (Zeiss FF450+), NIR-reflectance, and qAF (λ_ex_ = 488 nm), in both eyes. Quantitative AF technology includes an internal reference in the light path and allows valid comparison of RPE-attributed signal between baseline and follow-up and across patients [96]. Prior to capturing qAF images in 30° macular fields of both eyes we will bleach the retina, as recommended. On an exploratory basis for utility in fundus grading, we will take 50° posterior pole images with a white-light confocal device (Eidon, Centervue, Fremont, CA) for enhanced fundus detail in a 14.8 MB file format.

In terms of laboratory measures, plasma C-reactive protein (CRP), an acute-phase responder and sensitive indicator of systemic inflammation, will be quantified in an automated analyzer using turbidimetric reagent. Elevated CRP is associated with AMD, increases progression risk [97–99], and associates with delayed RMDA in persons with normal maculas [100]. Human choroid is immunoreactive for monomeric CRP [101–104], likely derived from circulating pentameric CRP (of hepatic origin) following irreversible dissociation after binding to damaged cells. CRP may be an effect modifier in associations between retinal structure and RMDA and scotopic sensitivity. DNA will be extracted to examine associations for *ARMS2* and *CFH* with visual function and retinal structure. We will examine the specificity of our recent finding that *ARMS2* and *CFH* are associated with delayed RMDA [17], using techniques previously described [17], by testing for associations with other types of scotopic and photopic dysfunction. Because visual tasks assess precise retinal mechanisms, the specificity of this relationship can potentially inform about the function of these high-risk alleles. For reasons elaborated in the Future Directions, we will also measure serum high-density lipoprotein (HDL) levels by standard enzymatic colorimetry assays and risk alleles for HDL genes associated with AMD risk and possibly involving systemic and intraocular mechanisms in lipid transfer [34].

Questionnaires are used to collect self-report information through interviewer-administration. Demographic information on birthdate, gender, race/ethnicity, education completed, and marital status is collected. General health information is collected by a questionnaire asking about the presence versus absence of 15 chronic medical conditions in the form of “Has a doctor ever told you that you have. ..? ” [100]. Participants are asked to bring a list of current prescription medications, over-the-counter medications, and nutritional supplements to their initial visit; these items are then inventoried by the coordinator. Questionnaires are also administered addressing alcohol use and smoking [100]. The Low Luminance Questionnaire (LLQ) [105] asks about difficulties that participants have in performing the visual activities of daily living under lower luminance or dim illumination conditions, including adapting to darkness and bright lights. Responses are organized into six subscales including driving, extreme lighting, mobility, emotional distress, general dim lighting and peripheral vision. The Vision Impairment in Low Luminance (VILL) questionnaire is also administered, which has content similar to that of the LLQ, focusing on visual difficulties under challenging lighting conditions. It is being used in the MACUSTAR study [106], an observational study on AMD being performed in Europe. We will examine the relationship between the VILL and LLQ in the ALSTAR2 sample. A cognitive screening test will be administered to obtain information on general cognitive status. The Montreal Cognitive Assessment (MOCA) [107] has been selected as a cognitive screener; it is designed for detection of mild cognitive dysfunction, possibly present in older adults recruited through eye clinics.

### Sample size determination and analysis plan

Each study aim will estimate the association between two continuous variables, visual function and structure (e.g., RMDA as measured by rod-intercept time and RPE thickness). Sample size requirements are based upon the calculation of correlation coefficients. Table 3 presents sample sizes required to have at least 80% power (alpha = 0.05) to detect a range of correlation coefficients as statistically significant. Because no published study uses specifically our proposed structure and function measures and our patient population, we used the literature reporting structure-function correlations ranging from 0.1–0.6 in aging and AMD to guide sample size estimation [86, 108–111]. Studies finding correlations > 0.3 typically address later stages of AMD (intermediate AMD and GA). Because we focus on early disease, we anticipate weaker structure-function relationships, ~ *r* = 0.2. Using these considerations as guides to compute sample size, 193 AMD and 193 normal patients are required in order to achieve statistical power of 80%. Given the longitudinal study design, we bolster this sample size for losses to follow-up over 3 years and the inability of some patients to perform some vision tests. Based on the ALSTAR1 experience [16, 71], we increase sample size by ~ 20% to address anticipated loss to follow-up. Thus, we enroll 240 each of persons in normal macula health and those with early AMD. We will also be including study of 20 young adults ages 20 to 30 years old to serve as a reference group, against which the older adults can be compared. They will participate in the same baseline protocol as the older adults.

**Table 3:** Sample Size Determination in ALSTAR2

Pearson correlation coefficient for alternative hypothesis	Sample size required
0.2	193
0.3	84
0.4	46

Statistical analyses will be conducted using SAS v. 9.4 (SAS Institute, Cary, NC) using a 2-sided alpha of 0.05. Although scientifically distinct, the 3 aims require similar analytical techniques, reflecting the underlying data structure being collected. A longitudinal study means that all primary measures will be collected at two points in time. However, for many measures, data will also be collected at specific retinal locations. Thus, statistical comparisons between structural and functional measurements must account for repeated measures and clustering of measurements within participants, i.e., eyes. The primary statistical tool to test hypotheses will be a mixed effects model. These models will be used to test both cross-sectional associations as well as prospective associations on changes in structure and function, both of which have hierarchical effects that need to be taken into account. The primary distinction between the models for cross-sectional and prospective associations is the inclusion of a time parameter in the latter. Prospective models will be used to explore the associations between joint changes in structural and functional measures but also whether baseline structural measures can predict functional changes. For example, considering the primary hypothesis for Aim 2A, i.e., RPE thickness is associated with scotopic dysfunction, the dependent variable will be RMDA and the main independent variables will be RPE volume (area x thickness across the macula) and mean normalized reflectivity. These measurements will be explored for their independent and joint effects on RMDA using unadjusted and adjusted models. For the cross-sectional analysis, the model will need to consider only the clustering of measurements within participants (i.e., at different locations). For the prospective analysis, a term must be added to the model to account for baseline and follow-up measurements.

It is important to note that the structure of secondary dependent variables (e.g., visual acuity) is heterogeneous with respect to clustering. For example, worse and better eye visual acuity is analyzed at the person level and therefore with person level independent variables, there is no need to account for clustering in the cross-sectional analyses; the prospective analyses will be akin to a repeated measures ANOVA model. The aforementioned analyses will all involve assessment of the confounding and/or modifying effects of demographic characteristics (e.g., age), behavioral characteristics (e.g., smoking) and biological measurements (e.g., CRP). It is important to note that the dearth of prior literature on structure-function relationships makes it difficult to a priori determine which measures may serve as confounders and/or effect modifiers. Further, the existing literature almost never takes these variables into account. However, based upon the extensive literature on risk factors for AMD incidence and progression, it is not unreasonable to consider characteristics such as smoking, chronic medical conditions, among others, as playing a role in these associations. Other variables such as age and whether eyes have progressed in AMD severity on the AREDS 9-step system will be addressed as confounder/effect modifier candidate variables. We will use multivariable analyses to evaluate the independent effects of the structural variables on slowed RMDA and other visual functions. These analyses will allow us to evaluate the relative extent to which each of the structural variables contribute to scotopic dysfunction. We will utilize standard techniques to evaluate the confounding and/or modifying effect of demographic and behavioral characteristics, seeking to identify structure-function relationships that are independent.

We will examine the relationship between self-reported vision problems (e.g., Low Luminance Questionnaire results) and structural and functional variables. These analyses will be structured the same as those described for the primary hypotheses. We will explore cross-sectional relationships as well as whether structural-functional changes over time are reflected in self-report measures. Although self-report measures are at the person-level, many of the structure-function measures, as described above, are clustered, thus for the simplest cross-sectional analyses, ANOVA will be used. For more complex cross-sectional and prospective analyses, mixed models will be employed.

## Discussion

A major barrier to developing treatments and prevention strategies for early AMD is a limited understanding of the temporal interrelationships among structural and functional characteristics during disease onset. Knowing which of AMD’s many layers is affected first can focus attention to treating or preventing problems and advising patients earlier than later in the course of AMD. A timeline can be generated for drusen-driven disease whereas SDD-driven disease is poorly understood [112, 113]. Existing large prospective studies focus on mechanisms of end-stage disease such as expansion of geographic atrophy, but little research addresses structural-functional mechanisms involved in disease onset. ALSTAR2 will fill in this gap. A related problem is even if a preventative strategy were developed, there are no valid, responsive functional endpoint measures for early AMD trials acceptable by both the FDA and the scientific community. The most promising structural endpoints will be those that correlate with function, facilitating their use as primary outcome measures. Thus, ALSTAR2 will enable the development of functionally valid, structural biomarkers for early AMD, suitable for use in forthcoming clinical trials as endpoint/outcome measures.

### Future directions, parallel studies, and testing of a center-surround hypothesis

In addition to collecting data for the three funded aims, we have efficiently managed participant burden to make time for data collection relevant to other scientific questions. Specifically, we will assess lens density on an individual basis for interpreting qAF measurements, since early reports indicate wide inter-individual variability due to lens opacity [114], and neither age-norms or statistical adjustment may give satisfactory answers. Thus, we have arranged for use of a slit lamp examination and digital camera image for each patient. We are also exploring means of using the already captured reflectance images to determine the absorption of blue light by the lens.

**Fig. 5:**
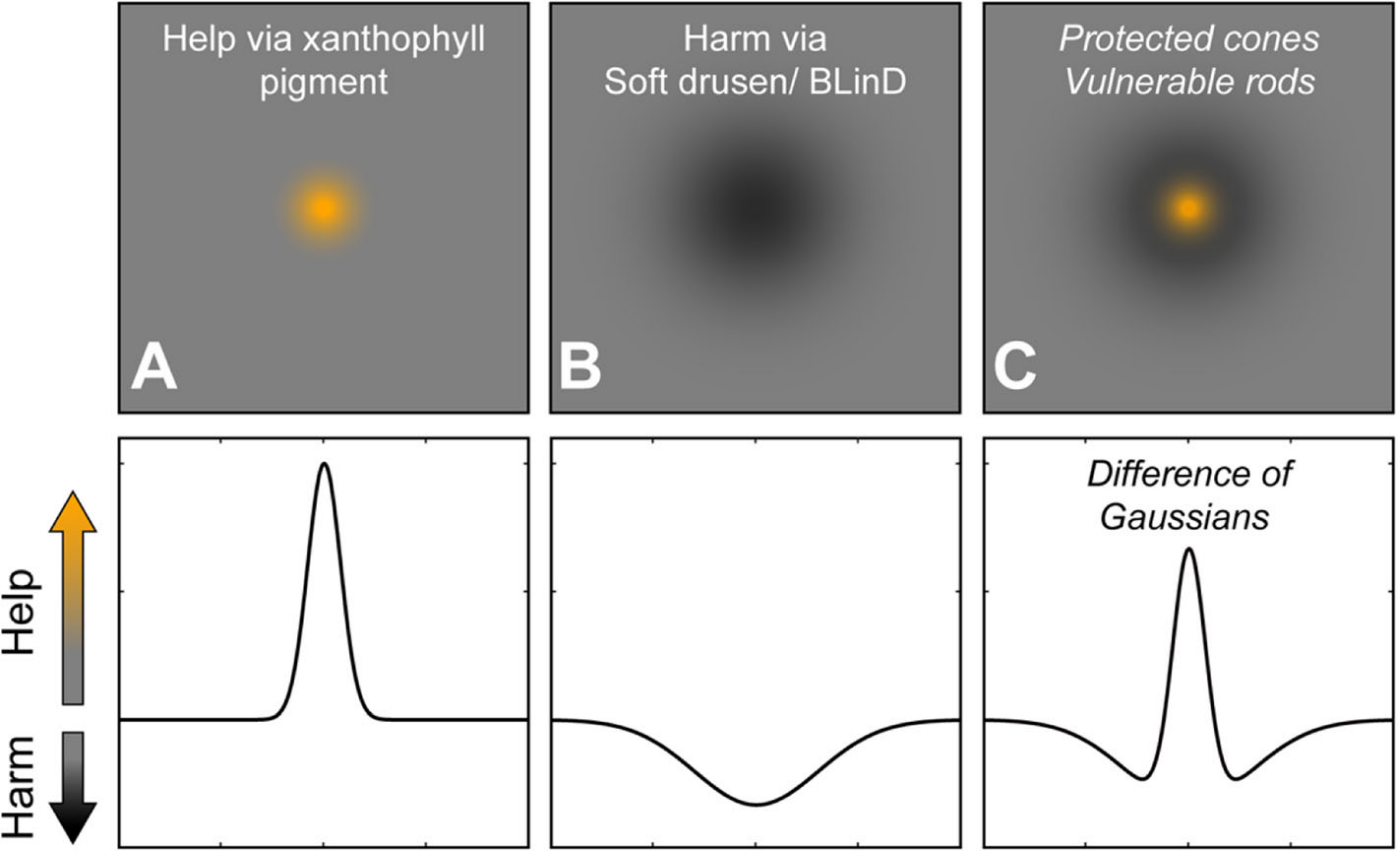
Center-surround model of cone resilience and rod vulnerability in aging and AMD. In the top row is an en face view of help via xanthophyll carotenoid pigment (orange) and harm via soft drusen/ basal linear deposit, as shown in Fig. 1. In the bottom row help and harm are plotted on one vertical axis, positive and negative directions, respectively. **a**. The distribution of xanthophyll carotenoids, as shown in Fig. 1, is a focused center of help in the central macula. **b** The distribution of soft druse material is shown as a broad circular area of harm (see Fig. 1). **c** Together, help and harm make a center of foveal cone resilience on top of a surround of para- and perifoveal rod vulnerability. The resemblance of this map to the maps of photoreceptor loss in human retinal aging in Fig. 1 are striking. See text for further details. Figure prepared with assistance of Deepayan Kar MS.

One goal in ALSTAR2 is to test and refine a hypothesis of AMD pathogenesis that we call the Center-Surround model of cone resilience and rod vulnerability in aging and AMD (Fig. 5). We seek to incorporate numerous data about vision in aging and AMD while also uniting the biology of drusen, neurobiology of the macula, and other aspects of human biology including genetics and epidemiology. In this model retinal topography is a powerful independent variable and a linkage to evolution, because the photoreceptors are distributed radially with strong eccentricity effects around the point of highest cone density. Evidence so far suggests that supporting cells in the outer retinal neurovascular unit are also distributed radially. Thus we will capture in addition to the planned visual function and imaging data described above, several other assessments. These include 2-wavelength autofluorescence images for macular pigment [115], serum for assays of lutein and zeaxanthin (macular pigment components), and sequence variants in the HDL genes from AMD genome-wide association studies [116]. The rationale behind these ancillary studies is two-fold: first is that xanthophyll macular pigment is a representative measure of Müller glia health and the capacity of these cells to support cone functions, including retinoid transfer, and second, that HDL-mediated xanthophyll transfer is the driving biologic process leading to the formation of high-risk soft drusen in central macula. We will next explicate the two elements of the Center-Surround model (Fig. 5) separately, center (fovea) first, then surround (para-, perifovea).

Longitudinal population-based studies have shown that drusen conferring risk for progression to late AMD are remarkably concentrated in the central macula [42–44, 117]. Drusen prevalence decreases by 2/3 from the central subfield of the ETDRS grid to the inner ring [42–44, 117], suggesting a basis in the biology of healthy fovea and raising four noteworthy points. First, it is interesting that cones are also highly concentrated in the central subfield and decrease in density by 2/3 in the inner ETDRS ring (Table 1). Second, Müller glia are concentrated in the foveal center and may equal cones in number [118, 119]. Third, the foveal center is rich in xanthophyll carotenoid pigments (lutein, zeaxanthin, meso-zeaxanthin) [120, 121], which could serve to protect the fovea. Fourth, Müller glia are newly indicated as major xanthophyll reservoirs by research on other glio-degenerative retinal diseases [121–126]. Thus, protection of foveal cones in aging and AMD can be in part attributed to the activity of Müller glia, a cell type frequently overlooked in AMD pathogenesis. The Center-Surround model (Fig. 5a) includes a sharp peak of protection due to xanthophyll carotenoids localized to foveal cones and Müller glia.

Because xanthophylls are present not only in the foveal center but also extend laterally into the perifovea, they also may play a role in forming the lipid-rich barrier that negatively impacts rods more than it does cones. Xanthophyll-rich macular tissue extends radially from the foveal center along the Henle fiber, inner plexiform, and nerve fiber layers [120, 127]. This distribution necessarily includes more than cones, long thought to be a major xanthophyll repository [128], because rods and outer trunks of Müller glia localize to the Henle fiber layer in addition to cones. Further, lateral extensions of Müller glia contribute to the plexiform and nerve fiber layers. Due to this topography, the transport of xanthophylls [129, 130] from choroid to the neurosensory retina must therefore cross a wider expanse of the ChC-BrM-RPE complex than just that directly underlying the foveal center (Fig. 2). Referring to drusen composition, the lipid-rich barrier in BrM comprises lipoproteins rich in cholesteryl ester released by RPE into BrM, and it, too, is thick under the fovea. These lipoproteins are the main constituent of soft drusen and a source of lipids that can be converted to pro-inflammatory and pro-angiogenic intermediates. The dominant fatty acid esterified to cholesterol in BrM lipoproteins is linoleate, the most abundant fatty acid in plasma. One of us (CAC) [34] proposed that plasma HDL delivering xanthophyll carotenoids and other lipophilic compounds to RPE for transfer to the retina is a plausible linoleate source. The RPE may thus strip HDL of xanthophylls and dispose of unneeded lipids in BrM where they are trapped as drusen precursors. This scenario could account for the concentration of soft drusen and their precursors in central macula. Thus the Center-Surround model (Fig. 2a) includes the lipid barrier in a broad low mound centered under the fovea, as wide as the extent of xanthophyll pigment in the neurosensory retina.

The center and surround can be combined as a difference of Gaussian curves. Figure 5 plots on a vertical Help/Harm axis, xanthophyll carotenoids as a focused center of help to cones (Fig. 5a) and drusen and their precursors as a broad valley of harm to rods (Fig. 5b). Combining these two effects creates a center of foveal cone resilience amidst an annular surround of para- and perifoveal rod vulnerability (Fig. 5c). The similarity of Fig. 5c to photoreceptor topography in human retinal aging (Fig. 1C-1) is striking. Rods are harmed as lipids accumulate in BrM, block exchange with the ChC, and promote drusen formation, inflammation, and type 1 neovascularization. Cones themselves are largely spared until late in AMD, because Müller glia protecting them are also supplied from retinal vasculature. Importantly, our model for the first time postulates a role in intra-ocular and systemic xanthophyll transfer for the HDL genes, the second largest AMD pathway after complement identified by genome-wide association studies. HDL genes are expressed in outer retina as well in liver and intestine, and RPE-specific deletion of at least one (ABCA1) impacts both cholesterol- and retinoid-handling pathways. Thus our study includes sequencing the HDL genes for AMD (apoE, CETP, LIPC, ABCA1) in patients for whom xanthophyll carotenoid abundance and topography and foveal morphology is determined by imaging. In our model Bruch’s membrane becomes lipid-laden and impacts rod function, due to high lipid trafficking (assessed by xanthophyll pigment abundance) and impaired egress to choriocapillaris (assessable by choriocapillaris density in aim 3), all governed by individual genetic predisposition. Data collected for ALSTAR2 will allow us to critically evaluate and refine this model.

## Data Availability

The data that is being generated in this study is stored at UAB. The data stripped of private health information identifiers are available from the corresponding author on reasonable request.

## Abbreviations

ALSTAR2: Alabama Study on Early Age-Related Macular Degeneration 2
AMD: Age-related macular degeneration
AREDS: Age-Related Eye Disease Study
BrM: Bruch’s membrane
CRP: C-reactive protein
FDA: Food and Drug Administration
GA: Geographic atrophy
HD: High-density lipoproteins
LLD: Low luminance deficit
LLQ: Low Luminance Questionnaire
MOCA: Montreal Cognitive Assessment
MPOD: Macular pigment optical density
SDOCT: Spectral domain optical coherence tomography
qAF: quantitative autofluorescence
RIT: Rod intercept time
RMDA: Rod-mediated dark adaptation
RPE: Retinal pigment epithelium
SDOCT: Spectral domain optical coherence tomography
S-MAIA: Scotopic Macular Integrity Assessment
SDD: Subretinal drusenoid deposits
UAB: University of Alabama at Birmingham
VILL: Vision Impairment in Low Luminance
